# Survival in Patients with Neuroendocrine Tumours of the Small Intestine: Nomogram Validation and Predictors of Survival

**DOI:** 10.3390/jcm9082502

**Published:** 2020-08-03

**Authors:** Sonja Levy, Linde M. van Veenendaal, Catharina M. Korse, Emilie C.H. Breekveldt, Wieke H.M. Verbeek, Menno R. Vriens, Koert F.D. Kuhlmann, José G. van den Berg, Gerlof D. Valk, Margot E.T. Tesselaar

**Affiliations:** 1 Department of Medical Oncology, Netherlands Cancer Institute, 1066CX Amsterdam, The Netherlands; l.v.veenendaal@nki.nl (L.M.v.V.); emilie.breekveldt@gmail.com (E.C.H.B.); m.tesselaar@nki.nl (M.E.T.T.); 2 Department of Clinical Chemistry, Netherlands Cancer Institute, 1066CX Amsterdam, The Netherlands; t.korse@nki.nl; 3 Department of Gastroenterology, Netherlands Cancer Institute, 1066CX Amsterdam, The Netherlands; w.verbeek@nki.nl; 4 Department of Endocrine Surgical Oncology, University Medical Centre Utrecht, 3584CX Utrecht, The Netherlands; mvriens@umcutrecht.nl; 5 Department of Surgical Oncology, Netherlands Cancer Institute, 1066CX Amsterdam, The Netherlands; k.kuhlmann@nki.nl; 6 Department of Pathology, Netherlands Cancer Institute, 1066CX Amsterdam, The Netherlands; j.vd.berg@nki.nl; 7 Department of Endocrine Oncology, University Medical Centre Utrecht, 3584CX Utrecht, the Netherlands; g.d.valk@umcutrecht.nl

**Keywords:** Neuroendocrine tumours, small-intestine, prognosis, nomogram, prediction model

## Abstract

Neuroendocrine tumours of the small intestine (SI-NETs) are rare and heterogeneous. There is an unmet need for prognostication of disease course and to aid treatment strategies. A previously developed nomogram based on clinical and tumour characteristics aims to predict disease-specific survival (DSS) in patients with a SI-NET. We aimed to validate the nomogram and identify predictors of survival. Four hundred patients with a grade 1 or 2 SI-NET were included, between January 2000 and June 2016. Predicted 5- and 10-year survival was compared to actual DSS. Multivariable analysis identified predictors for actual DSS. We found that in low-, medium- and high-risk groups 5-year nomogram DSS vs. actual DSS was 0.86 vs. 0.82 (*p* < 0.001), 0.52 vs. 0.71 (*p* < 0.001) and 0.26 vs. 0.53 (*p* < 0.001), respectively. Ten-year nomogram DSS vs. actual DSS was 0.68 vs. 0.69 (*p* < 0.001), 0.40 vs. 0.50 (*p* < 0.001) and 0.20 vs. 0.35 (*p* < 0.001), respectively. Age, WHO-performance score of 2, Ki-67 index ≥10, unknown primary tumour, CgA > 6x ULN and elevated liver tests were identified as independent predictors for a worse DSS. This shows that the nomogram was able to differentiate, but underestimated DSS for patients with a SI-NET. Improvement of prognostication incorporating new emerging biomarkers is necessary to adequately estimate survival.

## 1. Introduction

Neuroendocrine tumours (NETs) represent a heterogeneous group of rare tumours, most commonly presented in the gastrointestinal and bronchopulmonary tract [1,2]. The incidence of NETs is increasing, with a reported incidence of 6.61 per 100,000 individuals in 2011 [3,4]. NETs of the small intestine (SI-NETs) are, after pulmonary NETs, the second most common NETs and the most frequent malignancy of the small intestine [4,5,6,7]. Up to 73% of patients have metastases at time of diagnosis, predominantly in the liver [1,3,8,9]. Currently, the only potentially curative treatment for patients with SI-NETs consists of surgery [10,11]. Unfortunately, only a minority of patients (20–30%) with metastasised NETs are eligible for curative surgery [10,12,13]. In the palliative setting salvage surgery, surgery of the primary tumour, liver-directed therapies, somatostatin receptor analogues (SSAs) and peptide receptor radionuclide therapy (PRRT) are available [11,14,15]. Over the past decades, survival rates have increased, most likely due to the expanding therapeutic possibilities in the palliative setting and improved diagnostic techniques [4]. As a consequence, even though most patients present with metastatic disease, survival has been shown to be favourable, with a 5-year survival rate of 75% [16,17,18].

Predicting prognosis for an individual patient with a SI-NET remains challenging due to their heterogeneous disease course [19]. Several studies have identified prognostic factors mainly based on clinical and tumour characteristics. Nevertheless, their role in daily clinical practice remains limited [20,21,22]. Additionally, in recent years, the genomic landscape of SI-NETs has been under increasing investigation and identified several molecular prognostic factors [23,24]. However, these factors have not yet been widely implemented into clinical practice. Alternatively, the scientific focus has undergone a shift towards the development of ‘liquid biopsies’: blood-based biomarkers that can be used in clinical practice to predict disease presence or prognosis. These may constitute of circulating tumour cells (CTC), miRNA or circulating tumour transcripts. In several malignancies, liquid biopsies were able to predict prognosis [25,26,27,28]. In neuroendocrine tumours, both CTC and circulating tumour transcripts showed promising results for monitoring disease [29,30,31]. For instance, the presence of ≥one CTC in blood samples of 178 patients with NETs was shown to be independently associated with worse overall- and progression-free survival [30]. In a recent study of 152 patients with GEP-NETs, circulating tumour transcripts (NETest^®^), using a cut-off of 33%, have been shown to be the strongest predictor for disease progression [29]. Yet, often in these studies the value of clinical and tumour characteristics is underappreciated. Despite the identification of several prognostic factors and biomarkers, currently there remains an unmet need for adequate prognostication to predict disease course and survival for individual patients with SI-NETs.

In solid tumours, the classical method for prognostication has long been the tumour, node, metastasis (TNM) staging system [32]. Additionally, in SI-NETs, several pathological grading systems have been established over the past decades. The final adjustment to this system dates from 2017, wherein a reclassification has taken place of grade III neuroendocrine carcinoma (NEC) to well-differentiated grade III NET and poorly differentiated NEC [33]. However, both the TNM system and NET grading system fail to incorporate other possibly relevant factors for individual prognosis, including continuous variables such as age, or clinical variables such as performance status or gender. In this view, more elaborate statistical models for prognostication, i.e., medical nomograms, have been developed for several cancer types [34,35,36]. Nomograms have been shown to outperform the TNM staging system in predicting recurrence free- or disease-specific survival in several studies, demonstrating the clinical benefit of such models [35,36,37]. In 2010, Modlin et al. developed the first SI-NET nomogram based on clinical and tumour characteristics, to estimate an individual 5- and 10-year disease specific survival (DSS) [20]. Two studies have aimed to validate this nomogram for clinical use in daily practice. One study included 121 patients who underwent surgery with curative intent for a SI-NET; another validated the nomogram in 70 patients with a SI-NET [18,38]. To date, large validation studies have not been performed to assess the usefulness of the previously established nomogram and with that the value of clinical patient and tumour characteristics, in a real-world cohort of patients with a SI-NET with various stages of disease and treatment modalities.

We aim to assess whether prognostication based on this nomogram and the constituting clinical and tumour characteristics is suitable, especially considering the shifting focus to new emerging biomarkers. Therefore, in this study, we evaluate the prognostic ability of the nomogram in a large patient cohort treated in a European Neuroendocrine Tumour Society Centre of Excellence (ENETS CoE). In addition, prognostic predictors for survival were identified, which could contribute to further development of a prognostic model. 

## 2. Patients and Methods

### 2.1. Patients

All patients with a well-differentiated, grade I or grade II SI-NET referred to the Netherlands Cancer Institute (NCI) and University Medical Centre Utrecht (UMCU), an ENETS CoE, January 2000–June 2016, were included for retrospective analyses. To avoid misclassification of grade III NET/NEC, grade III NETs were excluded due to recent reclassifications in grading systems. Primarily, diagnosis was histopathology confirmed. When histopathological examination was not sufficient for a definitive diagnosis or in case of an unknown primary tumour, the consensus of a multidisciplinary expert panel was used to establish definitive diagnosis and assign the primary tumour type. Consensus was reached with the use of various parameters, such as elevated serum biomarkers: Chromogranin A (CgA), serotonin in thrombocytes or urinary 5-hydroxyindoleacetic acid (5-HIAA), typical desmoplastic fibrotic reaction in a mesenterial mass on imaging or functional symptoms referred to as the carcinoid syndrome (CS) or the presence of carcinoid heart disease (CHD). CS was considered flushing, diarrhoea and/or wheezing. CHD was confirmed with echocardiography. All relevant baseline and follow up characteristics were extracted from the longitudinal institutional neuroendocrine neoplasia database, which includes all patients treated in the centre. Since our centre functions as a tertiary referral centre, date of referral and consequently disease and clinical characteristics at time of referral were considered baseline for referred patients > 3 months after diagnosis. Urinary 5-HIAA and serotonin in thrombocytes levels > upper limit of normal (ULN) were combined into one variable: ‘elevated serotonin’, since urinary 5-HIAA was replaced by the latter in clinical follow up. Follow-up, vital status and cause of death were recorded. The study was conducted in agreement with the NCI/UMCU ethical guidelines and all patients gave consent for the use of their medical data as per institutional protocol.

### 2.2. Handling of Missing Variables

Missing values were predicted using multiple imputation. Variables that were assumed to be missing not at random were excluded from multiple imputation (tumour size, Ki-67, tumour grade, World Health Organisation (WHO) performance score and ethnicity). To establish patterns of missing values in the remaining variables Little’s missing completely at random (MCAR) was performed. CgA, serotonin and liver tests (including both liver function tests as liver enzymes) were found to be MCAR and were imputed using the fully conditional specification method. For imputation of continuous variables, a linear regression was used and for dichotomous variables logistic regression was used. The minimum amount of imputations was determined by the maximum percentage of missing data in the variables.

### 2.3. Nomogram and Prognostic Indicators

For all patients, individual predicted survival according to the nomogram was calculated. Nomogram survival reflects the predicted 5-year or 10-year DSS. For variables in which missing values were not imputed, best possible and worst possible scenario was created: missing values were assigned no points (scenario 1) or highest possible points (scenario 2), respectively. Hereafter, patients were divided in three equal strata: low-, medium- and high-risk stratum, according to their predicted survival probability. Actual DSS for these strata was calculated using Kaplan–Meier curves and was compared to the nomogram survival (for both scenarios 1 and 2) using paired signed rank test. DSS for the three risk strata were compared using the logrank test.

Additionally, the predictive value of the nomogram was evaluated in three patient categories. These categories were assumed to differ in a-priori survival probability: group 1 who underwent surgery with curative intent, group 2 who underwent surgery in a palliative setting, such as resection of primary tumour in metastatic setting or debulking surgery, and the final group 3 consisted of patients who were not eligible for surgical treatment. For these subgroups, nomogram survival was compared to actual DSS as well.

In our institutional patient cohort (both the original dataset as well as in the imputed dataset) a separate analysis to identify independent prognostic indicators for actual DSS was performed. 

### 2.4. Statistics

Variables were analysed using descriptive statistics: median with interquartile range (IQR) for continuous variables, frequency and percentage for categorical variables. DSS was calculated from date of diagnosis or date of referral for patients > 3 months after diagnosis. Patients alive before reaching one of the endpoints and patients who died of other causes were censored at their last time of follow-up or death, respectively. DSS and possible prognostic indicators were analysed using Kaplan–Meier curves, the logrank test and Cox’s proportional hazards regression. Variables with a *p*-value < 0.2 were included in multivariable analysis. Variable selection for multivariable analysis was performed using backward stepwise selection retaining variables with a *p*-value < 0.05. To avoid collinearity, the absence/presence of CS and CHD were combined in one variable. The same was done for tumour grade and Ki-67, combining these variables in grade I, grade II and <5% Ki-67, grade II and Ki-67 ≥ 5% but < 10% and grade II ≥ 10%. Statistical analyses were performed using IBM SPSS Statistics software, version 25.0.

## 3. Results

### 3.1. Patients

A total of 400 patients were included. Patient characteristics at baseline and nomogram variables can be found in Table 1. In the cohort, 192 patients (48%) were male and patients had a WHO performance status of 0, 1, and 2 in 161 (40%), 129 (32%) and 34 (9%) patients, respectively. Median age was 63 years (IQR 55–71). A total of 244 patients (61%) was referred within 3 months of diagnosis. Median time to referral for the remaining patients was 18 months (IQR 7–57). The ethnicity records were missing in 130 patients (33%). However, within the remaining 270 patients the majority (*n* = 253, 93%) was Caucasian, 7 patients (3%) were Black and 10 patients (4%) had another ethnicity. In 96 patients (24%) no primary tumour could be identified but consensus was reached by the multidisciplinary expert panel on the origin of the tumour. Most patients (*n* = 267, 67%) presented with functional symptoms. In 24 patients (6%) CHD was present at time of referral. Over three-quarters of patients (*n* = 305, 76%) had distant metastases at referral, of whom 236 patients (77%) had liver metastases. 

WHO grade I tumours accounted for the majority of patients (*n* = 265, 66%); grade II tumours were seen in 94 patients (24%). In 41 patients (10%), no distinction could be made between grade I and grade II. Nevertheless, these tumours were recognized as well-differentiated, low-grade tumours. Tumour size was determined by pathology reports and was available for patients that underwent surgery for their primary tumour (*n* = 138, 35%). Tumour size (cm) was <2, 2–2.5, 2.5–3 and >3 in 44 patients (11%), 22 patients (6%), 26 patients (4%) and 46 patients (12%), respectively. In approximately half of the patients (*n* = 202, 51%) Ki-67 index was <5%; 52 patients (13%) had a Ki-67 index between 5 and 10%, and 22 patients (6%) had a Ki-67 index ≥ 10%. After imputation, 107 patients (27%) had CgA levels > 6x ULN, 163 patients (41%) had elevated serotonin levels, and 35 patients (9%) had elevated liver tests.

### 3.2. Primary Treatment

In our cohort, 175 patients (44%) underwent surgery: 26 patients (7%) with curative intent and 149 patients (37%) with palliative intent. Nine patients (2%) underwent liver surgery for metastases. Somatostatin analogues were used by 152 patients (38%) whereas 21 patients (5%) were treated with nuclear- or radiotherapy, 4 patients (1%) were treated with liver embolization. 

### 3.3. Nomogram Survival and Actual DSS

**Figure 1:**
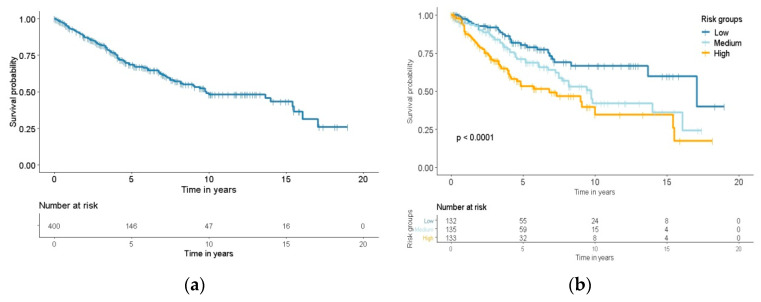
Kaplan–Meier curves for disease specific survival: (**a**) Disease specific survival curve of the institutional cohort; (**b**) Disease specific survival curves for low-, medium- and high-risk strata. Logrank test was performed for comparisons between survival.

**Figure 2:**
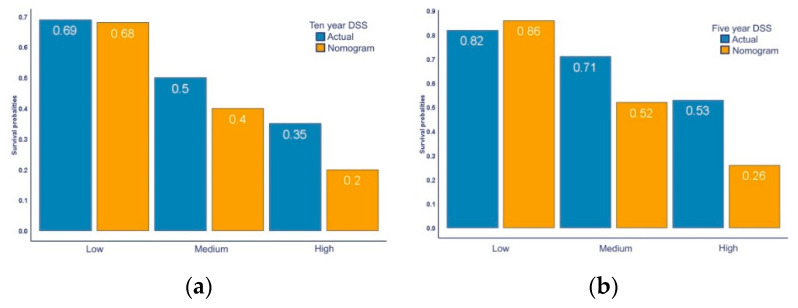
Actual disease specific survival vs. nomogram disease specific survival: (**a**) Five-year DSS categorised in low-, medium- and high-risk group, scenario 1; (**b**) Ten-year DSS categorised in low-, medium- and high-risk group, scenario 1.

Median follow up time was 5.0 years with a median actual DSS of 9.8 years for patients from the institutional cohort (Figure 1a). At the end of follow up, 80 patients (20%) died of their SI-NET, 50 patients (13%) died of an unknown cause of death, 21 patients (5%) died of other causes and the remaining 249 patients (62%) were alive at end of follow up. Considering the separate strata, median actual DSS was 17.1 in the low-risk group, 9.8 years in the medium-risk group, and 6.8 years in the high-risk group. The nomogram was able to differentiate between low-, medium- and high-risk groups (*p* < 0.001, Figure 1b). The predicted nomogram survival compared to the actual DSS for scenario 1 can be found in Figure 2a,b. In this scenario, patients in the low-, medium- and high- risk group had a 5-year predicted nomogram survival of 86%, 52% and 26%, compared to an actual 5-year DSS of 82%, 71% and 53%, respectively (Figure 2a, *p* < 0.001). The 10-year predicted nomogram survival was 68%, 40% and 20% compared to the actual 10-year DSS of 69%, 50% and 35% in the low-, medium- and high-risk group, respectively (Figure 2b, *p* < 0.001). Similar significant differences in DSS were seen for scenario 2 and in the different treatment subgroups. The nomogram overestimated 5-year DSS in the low risk group, but underestimated DSS in all other groups. The predicted nomogram survival for scenario 1 and 2 and actual DSS divided by subgroups can be found in the Supplementary Materials (Tables S1 and S2). 

The difference between nomogram survival and actual DSS ranged from 1% to 46%. The median difference was 24% (IQR 16–39) for 5-year survival and 20% (IQR 10–28) for 10-year survival. For all scenarios and treatment groups, the difference in predicted nomogram survival and actual DSS was the smallest for patients in the low-risk group, (median difference in survival 20%, IQR 1–29) compared to medium-risk (median difference in survival 22%, IQR 17–44) and high-risk group (median difference in survival 24%, IQR 9–36).

### 3.4. Prognostic Indicators

Univariable analysis identified age (HR 1.06), WHO performance score of 2 (HR 4.0), Ki-67 ≥ 10% (HR 1.3), grade 2 tumours (HR 1.8), an unknown primary tumour (HR 1.3), distant (HR 2.2) and liver metastases (HR 2.5), the presence of CS (HR 1.7) and CHD (HR 2.3), elevated CgA > 6 ULN (HR 4.3) and any elevated liver test (HR 4.9) to be associated with actual DSS (Table 2). In multivariable analysis age (HR 1.07), WHO performance status of 2 (HR 4.4), an unknown primary tumour (HR 3.2), Ki-67 index ≥ 10% (HR 12.6), CgA > 6 times ULN (HR 3.2) and elevated liver tests (HR 3.1) remained independent predictors for DSS in both the imputed as well as the non-imputed dataset. Results for multivariable analyses for both datasets can be found in Table 3.

## 4. Discussion

In this study, a previously designed nomogram, based on clinical and tumour characteristics, identified low-, medium- and high-risk groups in patients with SI-NETs. However, 5- and 10-year survival was underestimated for all scenarios and treatment groups, except for 5-year DSS in the low risk group. In our population, in multivariable analysis age, a WHO performance status of 2, an unknown primary tumour, Ki-67 index ≥ 10%, elevated CgA > 6x ULN, and elevated liver tests were the strongest independent predictors for a worse DSS. 

On the whole, predicted DSS by the nomogram was lower than the actual observed DSS. The low-risk subgroup for 5-year DSS in scenario 1 was the only subgroup where the opposite had occurred. This could be explained by the fact that in this scenario lowest possible nomogram scores were assigned to missing values in non-imputed variables, hence leading to an overestimation of DSS by the nomogram. The low-risk subgroup is the most susceptible to having assigned low nomogram points, because by definition, this group would have the highest DSS, and thus, the lowest nomogram score. This is illustrated by the fact that the difference between actual and nomogram DSS increases dramatically in scenario 2, resulting in poorer nomogram predicted DSS. Therefore, it is established that the nomogram underestimates actual DSS across all risk groups.

The evaluated nomogram is based on a large dataset from 7455 patients from the SEER database and variables were selected and weighed after extensive analyses of literature-curated data. However, the nomogram itself was initially validated in only 33 patients. Two earlier studies have attempted to validate the predictive properties of the SI-NET nomogram. Clift et al. (2017) showed that the predicted nomogram DSS matched the observed 5-year and 10-year DSS in a cohort of 70 patients [38]. This difference between our cohorts is not easily explained; our populations were quite comparable with regard to the baseline characteristics underlining the caution that should be taken by extrapolating findings from one population to another. However, patients were more often treated with PRRT (21% compared to 5% in our cohort), which suggests that their patients might have had more extensive disease burden.

In the study performed by Kelly et al. (2019), the nomogram was able to predict survival of patients in a cohort of 121 patients who underwent surgery with curative intent [18]. However, the nomogram score was not identified as an independent predictor for survival in multivariable analysis. The authors argued that this might be explained by their high survival rate of patients at the end of their follow up period (90.9%). Similarly, our patients had a significantly higher DSS than was estimated by the nomogram. Nevertheless, both Clift et al. and Kelly et al. recognized the prognostic potential of a nomogram based on clinical and tumour characteristics, with the need for extensive validation and possibly improvement. Subsequently, Kelly et al. recently developed a new nomogram for patients specifically from the United States [39].

Several studies have investigated survival of patients with NETs over different time periods and also found that survival increases in patients diagnosed in more recent time periods [3,4]. This is primarily due to new systemic treatment modalities that have emerged which have a beneficial effect on NET-related survival. SSAs made an entrance in 1987 and was the first systemic treatment option specifically for NETs. Initially, SSAs were found to reduce symptoms of carcinoid syndrome, but an antiproliferative effect was shown in the PROMID and CLARINET study [40,41,42]. PRRT was introduced in 2008. This treatment uses a radiolabelled somatostatin analogue to achieve local intratumoural nuclear therapy and showed a survival benefit for patients with a SI-NET treated with PRRT in the NETTER-1 study [43]. Additionally, diagnostic techniques for NETs have improved, earlier detection could contribute to an improved survival [4]. Likewise, the underestimation of survival by the nomogram could be explained by the fact that the nomogram was based on studies from 1997 and 2010 and on SEER data from patients diagnosed between 1977–2007. As a consequence, the nomogram was probably based on a poorer survival outcome compared to our cohort.

We found that a Ki-67 index ≥ 10% was associated with a worse DSS. This cut-off value is currently not used in clinical practice. The cut-off point most suited for distinguishing between grade I and II NET has been the subject of debate since the introduction of the Ki-67 index in the ENETS grading system in 2007. In a systematic review by Richards-Taylor et al. it was postulated that grade II and Ki-67 index ≤ 5% NET was more similar to grade I NET, compared to a grade II NET with Ki-67 index ≥ 5% [44]. Although in our cohort, a Ki-67 index < 5%, or Ki-67 index < 10% could not be identified as a separate prognostic factor for DSS, it does support the notion that the current Ki-67 index subdivision used for grading SI-NET might be insufficient for adequate prognostication. Future studies should aim to determine which cut-off values, if any, albeit in combination with other histopathological characteristics, would be more suitable.

An elevated CgA at referral of > 6x ULN was associated with a shorter DSS. Several studies have shown the prognostic value of baseline CgA as well [45,46,47]. This is in line with previous studies indicating that CgA is a marker of bulky disease, which is associated with poor survival [48]. Others have discussed that a change of CgA (of 25–20%) might be a better prognostic predictor than a single measurement, however this needs further validation [47,48]. Currently, there is a debate on the prognostic potential of CgA, since even in patients with metastatic disease, CgA is often within the normal range [29]. This suggests there might be room for improvement to decide the optimal threshold. 

Remarkably, liver metastases did not prove to be an independent prognostic predictor for DSS. Many other studies did show this association [7,49,50,51]. However, we included an unknown primary tumour as a separate variable in our multivariable analysis, which likely influenced this outcome since it overlaps with the presence of (liver) metastases. Yet, any elevation of liver test was associated with a worse DSS. It supports the notion that the extent of liver metastases (resulting in elevated liver test), might be more important than the presence of liver metastases alone [51,52].

A major strength of this study is the large patient population with detailed data on treatment and DSS. Moreover, our patient population treated in an ENETS CoE entails a representative patient population with a considerable follow up period incorporating all available therapies for patients with SI-NETs. Furthermore, we calculated the predicted nomogram survival and actual DSS for both best- and worst-case scenarios and included patients from all possible treatment categories. Additionally, to avoid confounding by indication, surgical treatment was excluded from analysis, since the policy in our centre is to perform a resection if technically achievable with either curative or palliative intention. Instead, nomogram survival and actual DSS was compared in different treatment groups. This ensures that the nomogram was evaluated in all possible real-world and clinically relevant patient groups, and assessed for its clinical validity in daily clinical practice.

Nevertheless, several limitations should be taken into consideration. Unfortunately, the included biomarkers were not always available because of changes in clinical practice over time. For example, CgA was measured from 2004, simultaneously abandoning urinary 5-HIAA as a biomarker, while serotonin in thrombocytes was introduced in 2010. However, the variable use of biomarkers is consistent with other studies involving NETs [11,53].

Another downside of a retrospective cohort study is missing data. This is inherent to collecting data in a longitudinal clinical database compared to data collection within the framework of a clinical trial. We handled missing data with the use of multiple imputation. By only imputing variables which we found to be completely at random, imputed values are not expected to be biased and statistical power could be maintained. Survival analyses were performed with an imputed and non-imputed dataset. For missing and non-imputed data, a best- and worst-case scenario was calculated assigning no points or the highest points possible. In both the imputed as non-imputed dataset and in various scenarios, our results were highly comparable. This suggests that, despite the retrospective character, our results are robust.

On the whole, while nomograms can fulfil an important role in personalized cancer care, when it accurately models clinical outcome [36], this nomogram in its current form and the clinical characteristics constituting the nomogram unfortunately appear insufficient to accurately predict individual prognosis. Recent advances in identifying (molecular) prognostic factors and the development of liquid biopsies such as CTC, or circulating tumour transcripts (the NETest^®^), appear to be a valuable addition for individualized prognostication [31,54]. Prognostic studies often focussed either solely on clinical and tumour characteristics [3,4,7,10] or the sole predictive value of a biomarker alone (subsequently abandoning the use of clinical and tumour characteristics) [29,53,55]. Our study confirms the prognostic potential of a nomogram based on clinical and tumour characteristics while underlining the need for improvement. Future studies should aim to combine clinical and tumour prognostic factors with potential new (molecular) biomarkers. By doing so, an advanced method of prognostic modelling for individual patients could be achieved.

## 5. Conclusions

A nomogram based on clinical data and tumour characteristics was the first extensive attempt for individual prognostication. The nomogram was able to differentiate between survival for patients in the low-, medium- and high-risk groups. However, the nomogram underestimated survival for 5- and 10-year survival for all but one scenario and all treatment groups. Age, WHO performance status of 2, an unknown primary tumour, Ki-67 index ≥ 10%, elevated CgA > 6x ULN, and elevated liver tests were identified as independent predictors for a worse DSS. Our findings imply that improvement of individualized estimation of prognosis is desirable. Future studies should aim to combine clinical and tumour prognostic factors with potential new (molecular) biomarkers.

## Figures and Tables

**Table 1:** Baseline characteristics for the institutional cohort and imputed variables.

Patient Characteristics	*n* (%) or Median (IQR)	Imputed *n* (%)
Sex		
Male	192 (48)	
Female	208 (52)	
Age at baseline *	63 (55–71)	
WHO PS		
0	161 (40.2)	
1	129 (32.3)	
2	34 (8.5)	
Missing	76 (19)	
Ethnicity *		
Caucasian	253 (63.2)	
Black	7 (1.8)	
Other	10 (2.5)	
Missing	130 (32.5)	
Primary tumour		
SI	304 (76.0)	
Unknown primary	96 (24.0)	
Carcinoid syndrome *	267 (66.8)	
CHD *	24 (6.0)	
Distant metastases at baseline	305 (76.3)	
Liver metastases at baseline *	236 (59.0)	
Tumour grade *		
1	265 (66.2)	
2	94 (23.5)	
Unknown	41 (10.3)	
Tumour size *		
<2	44 (11.0)	
2–2.5	22 (5.5)	
2.5–3	26 (3.8)	
>3	46 (11.5)	
Missing	127 (31.8)	
Max Ki-67 index *		
<5	202 (50.5)	
<10	52 (13.0)	
≥10	22 (5.5)	
Missing	124 (31.0)	
Elevated CgA >6x ULN *	98 (24.5)	107 (26.8)
Missing	88 (22.0)	
Elevated serotonin *	143 (35.8)	163 (40.8)
Missing	212 (53.0)	
Elevated liver tests *	33 (8.3)	35 (8.8)
Missing	53 (13.3)	
**Treatment**		
Groups		
1. Surgery with curative intent	26 (6.5)	
2. Surgery with palliative intent	149 (37.3)	
3. No surgery	225 (56.2)	
SSAs *	152 (38.0)	
Surgery	175 (43.8)	
Liver surgery *	9 (2.3)	
PRRT/RT	21 (5.3)	
Embolization	4 (1.0)	

* Nomogram variable; WHO PS: World Health Organisation Performance Score; G1: grade 1; G2: grade 2; SI: small intestine; UP: unknown primary; CS: carcinoid syndrome; CHD: carcinoid heart disease; CgA: chromogranin A; liver tests: any elevation of alkaline phosphatase, gammaglutyltransferase or bilirubin; ULN: upper limit of normal; SSAs: somatostatin analogues; PRRT: peptide receptor radionuclide therapy; RT: radiotherapy.

**Table 2:** Univariable analysis for disease specific survival.

Variable	HR	*p*	CI
Age	1.06	**<0.001**	1.04–1.08
Gender	0.72	0.067	0.51–1.02
Ethnicity			
Caucasian	1		
Black	1.11	0.882	0.27–4.55
Other	1.01	0.992	0.25–4.12
WHO PS			
0	1		
1	1.72	**0.014**	1.11–2.67
2	4.03	**<0.001**	2.27–7.14
Grade			
1	1		
2	1.83	**0.003**	1.23–2.74
Ki-67 index			
G1	1		
G2			
<5	1.00	0.994	0.46–2.16
<10	1.28	0.429	0.69–2.36
≥10	4.0	**<0.001**	2.16–7.40
Primary			
SI	1		
Unknown primary	1.34	**<0.001**	1.23–1.46
Distant metastases	2.23	**0.001**	1.39–3.68
Liver metastases	2.51	**<0.001**	1.70–3.70
CS	1.72	**0.011**	1.13–2.60
CHD	2.27	**0.002**	1.34–3.83
CgA > 6x ULN	4.28	**<0.001**	2.83–6.49
Serotonin > ULN	1.07	0.814	0.59–1.94
Liver tests > ULN	4.94	**<0.001**	2.92–8.36

WHO PS: World Health Organisation Performance Score; G1: grade 1; G2: grade 2; SI: small intestine; UP: unknown primary; CS: carcinoid syndrome; CHD: carcinoid heart disease; CgA: elevated chromogranin A; liver tests: any elevation of alkaline phosphatase; gammaglutyltransferase or bilirubin. ULN: upper limit of normal.

**Table 3:** Multivariable analysis for disease specific survival in original dataset and imputed dataset.

	Original Dataset			Imputed Dataset		
Variable	HR	*p*	CI	HR	*p*	CI
Age	1.07	**<0.001**	1.04–1.10	1.07	**<0.001**	1.04–1.09
WHO PS						
0	1			1		
1	1.11	0.756	0.58–2.14	1.13	0.676	0.62–2.07
2	4.40	**<0.001**	2.09–9.20	4.14	**<0.001**	2.01–8.51
Ki-67 index						
G1	1			1		
G2						
<5	2.38	0.121	0.79–7.11	2.51	0.067	0.94–6.73
<10	1.04	0.918	0.46–2.36	0.99	0.981	0.0.44–2.22
≥10	12.61	**<0.001**	5.51–28.84	11.56	**<0.001**	5.15–25.93
Primary						
SI	1			1		
UP	3.24	**<0.001**	1.42–4.74	2.32	**0.003**	1.32–4.01
CgA > 6x ULN	3.24	**<0.001**	1.91–5.46	3.43	**<0.001**	2.06–5.7
Liver tests > ULN	3.10	**0.004**	1.45–6.63	3.12	**0.003**	1.46–6.60

WHO PS: World Health Organisation Performance Score; G1: grade 1; G2: grade 2; SI: small intestine; UP: unknown primary; CgA: elevated chromogranin A; liver tests: any elevation of alkaline phosphatase; gammaglutyltransferase or bilirubin. ULN: upper limit of normal.

## Supplementary Materials

The following are available online at https://www.mdpi.com/2077-0383/9/8/2502/s1, Table S1: Nomogram survival and actual DSS, Table S2: Nomogram survival and actual DSS in the three treatment groups.
