# Supplementary material for: Survival in Patients with Neuroendocrine Tumours of the Small Intestine: Nomogram Validation and Predictors of Survival
Source: J Clin Med. 2020 Aug 3;9(8):2502. doi: 10.3390/jcm9082502 (PMC7464451; doi:10.3390/jcm9082502)
Supplement: Supplementary file 1 [file jcm-09-02502-s001.pdf]

Supplementary material

Table 1. Nomogram survival and actual DSS.

Stratum	Actual 5Y DSS	Nomogram 5YDSS	P	Actual 10Y DSS	Nomogram 10YDSS	P
			Scenario 1			
Low	0.82	0.86	<0.001	0.69	0.68	<0.001
Medium	0.71	0.52	<0.001	0.50	0.40	<0.001
High	0.53	0.26	<0.001	0.35	0.20	<0.001
			Scenario 2			
Low	0.89	0.64	<0.001	0.79	0.50	<0.001
Medium	0.70	0.25	<0.001	0.46	0.20	<0.001
High	0.47	0.08	<0.001	0.29	0.08	<0.001

Stratum: column indicating low-, medium- or high risk groups based on nomogram scores; 5Y DSS: five year disease specific survival, 10Y DSS: 10-year disease specific survival.

Table 2. Nomogram survival and actual DSS.

	Actual 5Y DSS	Nomogra m 5YDSS	P	Actual 10Y DSS	Nomogram 10YDSS	P
			Scenario 1			
			Curative surgery			
Low	0.96	0.88	<0.001	1	0.70	<0.001
Medium	1	0.56	<0.001	1	0.43	<0.001
High	1	0.33	<0.001	0.67	0.23	<0.001
			Palliative surgery			
Low	0.82	0.86	<0.001	0.78	0.68	<0.001
Medium	0.67	0.49	<0.001	0.46	0.38	<0.001
High	0.50	0.26	<0.001	0.33	0.20	<0.001
			No surgery			
Low	0.81	0.85	<0.001	0.69	0.68	<0.001
Medium	0.68	0.52	<0.001	0.49	0.40	<0.001
High	0.50	0.26	<0.001	0.36	0.19	<0.001
			Scenario 2			
			Curative surgery			
Low	0.93	0.69	<0.001	1	0.54	<0.001
Medium	1	0.23	<0.001	1	0.16	<0.001
High	1	0.08	<0.001	0.67	0.08	<0.001
			Palliative surgery			
Low	0.83	0.67	<0.001	0.78	0.53	<0.001
Medium	0.67	0.24	<0.001	0.42	0.18	<0.001
High	0.50	0.08	<0.001	0.36	0.08	<0.001
			No surgery			
Low	0.91	0.59	<0.001	0.74	0.45	<0.001
Medium	0.69	0.23	<0.001	0.36	0.18	<0.001
High	0.45	0.08	<0.001	0.30	0.08	<0.001

Stratum: column indicating low-, medium- or high risk groups based on nomogram scores. 5Y DSS: 5-year disease specific survival, 10Y DSS: 5-year disease specific survival.
